# A retrospective review of oral low-dose sirolimus (rapamycin) for the treatment of active uveitis

**DOI:** 10.1007/s12348-010-0015-5

**Published:** 2010-12-07

**Authors:** Brandon N. Phillips, Keith J. Wroblewski

**Affiliations:** Ophthalmology Service, Walter Reed Army Medical Center, 6900 Georgia Avenue NW, Washington, DC 20307 USA

**Keywords:** Sirolimus, Rapamycin, Uveitis, Inflammation, Immunomodulatory, Corticosteroid-sparing agent

## Abstract

**Purpose:**

The purpose of this study is to elicit the role of oral low-dose sirolimus as a corticosteriod-sparing agent for active uveitis.

**Methods:**

A retrospective, interventional case series was performed by reviewing the clinical records of all patients treated with oral, low-dose sirolimus (1–4 mg daily) for severe uveitis. Data reviewed included symptomatic improvement, Snellen best-corrected visual acuity, corticosteroid requirement, sirolimus levels, intraocular inflammation, spectral-domain optical coherence tomography, and fluorescein angiogram. Primary outcome measures were determined by the ability to decrease the intraocular inflammation, corticosteroid requirement, and frequency of flares.

**Results:**

Eight patients with varied diagnoses were treated with oral low-dose sirolimus for severe, chronic uveitis between 2008 and 2010. In four of the eight patients, there was an improvement of all primary outcome measures. While sirolimus monotherapy was successful in only one patient, a sirolimus/methotrexate combination was successful in three patients. Although there was a good initial response in three patients, treatment was a failure after serious side effects forced the cessation of sirolimus therapy. One patient was lost to follow-up.

**Conclusion:**

Sirolimus may have a limited role in severe uveitis as an adjunct corticosteroid-sparing agent in combination with more standard immunosuppressive agents. Oral low-dose sirolimus appeared to be better tolerated than higher doses, but there were a significant number of adverse events, requiring therapy to be stopped.

## Introduction

Uveitis results from a wide variety of infectious and non-infectious insults, and it often presents a difficult challenge in terms of etiology and treatment. It is estimated that more than 280,000 people in the US are affected by uveitis each year, and it is responsible for up to 10% of all cases of blindness [1]. Patients can become unresponsive or refractory to topical or oral corticosteroid therapy, requiring other classes of immunomodulatory drugs during the course of their treatment. Methotrexate, cyclosporine, azathioprine, and mycophenolate mofetil are the more common corticosteroid-sparing agents currently used to treat uveitis; however, these agents are limited by a narrow therapeutic window and have significant side effects that often modulate their use.

Sirolimus, also known as rapamycin, inhibits antigen-induced proliferations of T cells and B cells and antibody production by inhibiting mTOR, a key regulatory enzyme required for proliferation and differentiation [2]. Sirolimus forms a complex with the intracellular protein FKBP12, which blocks the activation of the cell cycle kinase mTOR, inhibiting the multifunctional serine–threonine kinase, resulting in the blockage of progression at the juncture of the G1 and S phases [3, 4]. As an mTOR inhibitor, sirolimus has a broad spectrum of activity that has demonstrated the ability to inhibit inflammation, proliferation, angiogenesis, fibrosis, and hyperpermeability. Sirolimus currently has multiple uses in the prevention of rejection in organ transplantation and, recently, in the treatment of advanced renal cell carcinoma [5]. Sirolimus-eluting cardiac stents have been shown to limit the rate of overgrowth of tissue and thus prevent coronary restenosis [6]. Early studies suggest that it may be an effective agent for controlling severe uveitis and that it may also have a role in treating age-related macular degeneration [7].

## Methods

A retrospective chart review of all electronic medical records of patients treated with sirolimus for severe uveitis at the Walter Reed Army Medical Center Ophthalmology Service was completed. The purpose of this study was to elicit the role of low-dose oral sirolimus as a corticosteriod-sparing agent in the treatment of active uveitis. Permission was granted by the Institutional Review Board for this research, and this research adhered to the tenets of the Declaration of Helsinki. All patients had previously had an extensive workup to include laboratory examination, radiologic studies, and biopsy, if necessary. The etiology of the disease was varied, and they are listed in Table 1.

**Table 1:** Patient's demographics, clinical details, and previous treatments

Case	Age	Sex	History	Diagnosis	Previous therapy	Reason for sirolimus
1	67	M	Panuveitis OU with CME OD > OS treated with topical steroids.	Sarcoidosis	Topical ketorolac, topical prednisolone	Patient wanted to avoid oral prednisone
2	39	M	Bilateral recurrent anterior uveitis and scleritis treated with multiple orbital floor steroid injections.	Sarcoidosis	Subtenon triamcinolone, oral prednisone	Frequent flares
3	31	F	Chronic anterior uveitis with CME OD previously treated with prednisone and methotrexate. Poor control of inflammation with frequent flares.	Idiopathic	Subtenon triamcinolone, oral prednisone, oral methotrexate	Uncontrolled inflammation with previous regimen
4	26	M	Bilateral chronic intermediate uveitis. Treated for Lyme disease by infectious diseases.	Secondary to Lyme disease	Oral prednisone, oral methotrexate	Uncontrolled inflammation with previous regimen
5	54	M	Granulomatous anterior uveitis with vasculitis and choroidal infiltrates OD s/p repair of ruptured globe and eventual enucleation OS.	Sympathetic ophthalmia	IV methylprednisolone, oral prednisone	Uncontrolled inflammation despite maximal dose of oral prednisone
6	61	M	Panuveitis OU treated with orbital floor steroid injections and high-dose oral corticosteroids. History of hepatitis C and aseptic meningitis.	Sarcoidosis	Subtenon triamcinolone, oral prednisone	Avoid higher doses of oral prednisone
7	79	F	Panuveitis with CME OD > OS treated with oral corticosteroids. History of chronic anemia.	Idiopathic	Oral prednisone	Avoid higher doses of oral prednisone
8	21	M	Recurrent panuveitis treated with high doses of oral corticosteroids.	HLA B27 positive	Oral prednisone	Uncontrolled inflammation despite maximal dose of oral prednisone

*F* female, *M* male

A thorough risk–benefit analysis and discussion of immunomodulatory agents and corticosteriod therapy were had with each patient. Every patient started on sirolimus chose it over a more standard regimen. Patients were started on sirolimus 1–4 mg po daily. Sirolimus blood levels were drawn serially, and sirolimus was titrated to 4–12 ng/mL. For most indications, the target serum blood level of sirolimus is 10–20 μg/mL. Both experimental animal and clinical data suggest that adverse events and their associated severity are correlated with blood concentrations [2]. Therefore, low-dose oral sirolimus with a target serum blood level of 4–12 μg/mL should be better tolerated. Patients were monitored regularly to determine effectiveness of the therapy and side effect profiles. Data reviewed included symptomatic improvement, Snellen best-corrected visual acuity, corticosteroid requirement, sirolimus levels, intraocular inflammation determined with slit lamp examination and binocular indirect ophthalmoscopy using standardization of uveitis nomenclature criteria [8], Cirrus spectral-domain optical coherence tomography, and fluorescein angiograms. Primary outcome measures were determined by the ability to decrease the intraocular inflammation, corticosteroid requirement, and frequency of flares.

## Results

Eight patients were treated with oral low-dose sirolimus for active uveitis between 2008 and 2010. The patients' demographics, clinical details, and previous treatments are listed in Table 1. Three patients had a diagnosis of sarcoidosis, two patients had idiopathic uveitis, one patient had sympathetic ophthalmia, one patient had HLA-B27 associated panuveitis, and one patient had intermediate uveitis secondary to Lyme disease. There were six males and two females. The ages ranged from 21 to 79 with a mean age of 47. Seven of eight patients were previously treated with systemic corticosteroids to control their disease at the initiation of oral, low-dose sirolimus. One patient was started on sirolimus as a first-line agent. Three patients had previously been treated with a subtenons injection of triamcinolone. One patient had been previously treated with a corticosteroid-sparing agent, and one was treated with intravenous methylprednisolone.

The clinical outcomes are listed in Tables 2 and 3. In four of the eight patients, oral low-dose sirolimus was considered a success. The duration of treatment ranged from 21 to 65 weeks, with a mean duration of 44.5 weeks. Patient 1 had an improvement of all primary outcome measures with oral low-dose sirolimus monotherapy. In contrast, patients 2–4 had improvements of all primary outcome measures with a combination of oral low-dose sirolimus and methotrexate. In patients 2 and 3, methotrexate was added to the sirolimus regimen after a flare. Patient 3 had previously failed methotrexate therapy. In patient 4, oral low-dose sirolimus was added to the methotrexate regimen after a flare.

**Table 2:** Clinical outcomes

Case	Pre-sirolimus BCVA	Post-sirolimus BCVA	Clinical course	Corticosteroid requirement	Clinical outcome	Current therapy
1	OD–20/50	OD–20/25	Resolution with sirolimus 2 mg/day	Did not require prednisone	Decreased inflammation	Sirolimus 2 mg/day
	OS–20/25	OS–20/20			Decreased flare rate	
2	OD–20/30	OD–20/15	Initial improvement with a flare 5 weeks after starting sirolimus	40 to 5 mg/day with sirolimus/MTX combination	Decreased inflammation	Sirolimus 3 mg/day
	OS–20/30	OS–20/20	No additional flares after adding MTX		Decreased flare rate	MTX 20 mg/week
						Prednisone 5 mg/day
3	OD–20/150	OD–20/25	No initial improvement with sirolimus 2 mg/day	40 to 5 mg/day with sirolimus/MTX combination.	Decreased inflammation, decreased flare rate	Sirolimus 4 mg/day
			Increased to 4 mg/day and restarted MTX 10 mg/week with resolution of CME			MTX 10 mg/week
						Prednisone 5 mg/day
4	OD–20/25	OD–20/20	Improvement with the addition of sirolimus 3 mg/day to MTX 25 mg/week	10 mg/day to tapered off	Decreased inflammation	Sirolimus 3 mg/day
	OS–20/25	OS–20/20			Decreased flare rate	MTX 25 mg/week
5	OD–20/20	OD–20/15	Improvement with sirolimus 3 mg/day and cyclosporine 50 mg BID	20 to 10 mg/day while on sirolimus	Failure due to side effects	Cyclosporine 100 mg BID
			Sirolimus was stopped after the patient developed a DVT			Prednisone 20 mg/day
6	OD–20/30	OD–20/25	Initial improvement of vision but sirolimus was stopped due to a recurrence of aseptic meningitis	Not enough data	Failure due to side effects	Prednisone 20 mg/day
	OS–20/30	OS–20/20				
7	OD–20/60	No data	Platelet count dropped from 160,000 to 80,000 after starting sirolimus	Not enough data	Failure due to side effects	Prednisone 20 mg/day
	OS–20/50		Sirolimus was stopped and the platelets rebounded			
8	OD–20/150	No data	Missed several follow-up appointments	No data	Lost to follow-up	No data
	OS–10/100					

*MTX* methotrexate; *BCVA* best corrected visual acuity; *CME* cystoid macular edema

**Table 3:** Duration of sirolimus therapy and dosage

Case	Duration of therapy (weeks)	Initial dose (mg)	Final dose (mg)	Weight (kg)	Number of flares	Still taking	Side effects
1	65	2	2	95	0	Yes	No significant side effects to date
2	50	3	4	122	1	Yes	No significant side effects to date
3	42	2	4	86	1	Yes	No significant side effects to date
4	21	3	3	102	0	Yes	No significant side effects to date
5	8	2	3	79	1	No	DVT-left popliteal
6	6	2	2	72	0	No	Nausea, vomiting, recurrence of aseptic meningitis
7	4	2	2	64	0	No	Thrombocytopenia
8	2	2	2	80	No data	No	No data

Treatment was considered a failure in three patients after serious side effects forced the cessation of sirolimus. Patient 5 was found to have a left popliteal deep vein thrombosis during week 8 of sirolimus. It was later discovered that the patient smoked more significantly than initially reported (one pack per day). Patient 6 developed mental status changes due to a recurrence of aseptic meningitis during week 6 of sirolimus. The patient had a history of hepatitis B and one previous episode of aseptic meningitis. The patient recovered with no sequelae. Patient 7 developed thrombocytopenia during week 4 of sirolimus. Her platelet count dropped from 160,000 to 80,000 and rebounded after cessation. However, patients 5 and 6 did have a good initial response to oral low-dose sirolimus therapy. Patient 8 was lost to follow-up.

## Discussion

Sirolimus is a unique and potent agent with broad anti-inflammatory and immunomodulatory activities. Early studies suggest sirolimus may have many diverse roles, including treating patients with retinal thickening secondary to leakage as in neovascular age-related macular degeneration and diabetic macular edema [9]. Sirolimus inhibits the translation and activity of hypoxia-inducible factor-1 alpha (HIF-1a), a stress-activated protein that is a potent stimulator of vascular endothelial growth factor (VEGF), and its inhibition affects VEGF both upstream at the production level and downstream at the receptor level [10]. This inhibition is a direct effect on the endothelial barrier, independent of vasodilation, and plays an important role in angiogenesis and hyperpermeability [11]. A study showed that sirolimus was superior to VEGF inhibition in a co-culture assay of endothelial cells (EC) and retinal pigment epithelium (RPE) by reducing EC sprouting in groups that did not respond to anti-VEGF treatment and by reducing both VEGF production in RPE and the responsiveness of EC to stimulation [12]. Sirolimus might therefore be a therapeutic option for choroidal neovascularization (NSV) patients that do not respond sufficiently to the established anti-VEGF treatments or may be an excellent combination agent, improving overall outcomes and the intervals between anti-VEGF injections. Sirolimus also has broad inhibitory effects of many inflammatory factors that are not currently addressed in treatment relevant to the development of geographic atrophy, such as fibrosis, leading to irreversible photoreceptor death. It was also reported that, following the administration of sirolimus, there was a reduction of the tumor burden in LH_BETA_T_AG_ transgenic retinoblastoma tumors [Murray TG, Piña Y, Houston S, et al. Retinoblastoma Tumor Burden Control: Periocular mTOR Inhibitor Rapamycin Decreases Tumor Burden in Advanced LH_BETA_T_AG_ Murine Retinoblastoma. Presented at: Annual Meeting of Association for Research in Vision and Ophthalmology (ARVO); May 2010; Fort Lauderdale, Fla].

The role of sirolimus in treating patients with severe uveitis is evolving. Early studies suggest sirolimus may be an effective agent for controlling uveitis, but its role was not clearly defined [Wroblewski KJ, Sen HN, Yeh S, it al. Combination Daclizumab and Sirolimus Therapy for the Induction of Immune Tolerance in Non-Infectious Intermediate and Posterior Uveitis. Paper presented at: ARVO; May 2009; Fort Lauderdale, Fla]. Two experimental studies using animal models suggested a synergistic therapeutic effect of combination therapy with low doses of calcineurin inhibitors cyclosporine and tacrolimus [13]. A study of human patients with refractory uveitis, limited to patients whose disease was not controlled with at least two or more separate steroid immunosuppressants, showed sirolimus therapy was effective in five of eight patients [14]. Treatment in three patients was considered a failure due to intolerable side effects or failure to control uveitis. Maximum doses ranged from 4 to 12 mg/day, with blood levels of up to 25 ng/mL. Researchers found that higher blood levels led to an increased side effect profile, mostly dermatological and gastrointestinal in nature, with little additional benefits.

In our series, three out of seven patients stopped taking sirolimus due to a serious side effect. However, two of those patients had significant confounding factors. The other four patients tolerated the medication well with little to no side effects. This could be explained by the relatively low doses of sirolimus used in comparison to the previous study. As a solo immunosuppressive agent, our limited study suggests that oral low-dose sirolimus has little role in the treatment of active uveitis. However, oral low-dose sirolimus used in combination with methotrexate worked well for three patients in this series. Commonly described side effects include anemia, thrombocytopenia, hypercholesterolemia, arthralgias, and gastrointestinal problems. Sirolimus has also been shown to be associated with Bk virus-associated nephropathy, although at the lower end of the range described with various other contemporaneous immunosuppressive regimens [15].

Since oral or intravenous administration presents the issues of systemic immunosuppression and side effects, investigators have been evaluating the safety, tolerability, and biological activity of sirolimus when delivered by either a subconjunctival or intravitreal injection, making systemic exposure negligible. Animal studies have shown that there is no retinal toxicity with intravitreal sirolimus injections [16, 17]. It was recently suggested that retinal and choroidal sirolimus levels are sustained longer, following subconjunctival injection, as the sclera acts as a reservoir for the highly lipophilic drug [Nivaggioli T, Bao JX, Farooq S, et al. Pharmacokinetics of a locally administered subconjunctival ocular formulation of sirolimus in rabbits and humans. Paper presented at: ARVO; May 2009; Fort Lauderdale, Fla]. There were no dose-limiting toxicities, ocular inflammation, or increase in intraocular pressure.

## Conclusion

Oral low-dose sirolimus appears to have a limited role in active uveitis as an adjunct corticosteroid-sparing agent in combination with more standard immunosuppressive agents. Oral low-dose sirolimus appeared to be better tolerated than higher doses, but there were a significant number of adverse events, requiring therapy to be stopped. The study suffers from limited numbers and a selection bias. Sirolimus should only be used after proper patient selection with an extensive risk–benefit analysis and a thorough discussion with the patient. Patients taking sirolimus need close monitoring, including necessary lab work such as blood levels, CBCs, liver function tests (LFT), and lipid levels.
